# Survival strategies of aerobic methanotrophs under hypoxia in methanogenic lake sediments

**DOI:** 10.1186/s40793-024-00586-1

**Published:** 2024-07-02

**Authors:** Almog Gafni, Maxim Rubin-Blum, Colin Murrell, Hanni Vigderovich, Werner Eckert, Nasmille Larke-Mejía, Orit Sivan

**Affiliations:** 1 Department of Earth and Environmental Sciences, Ben-Gurion University of the Negev, Beer Sheva, Israel; 2 Israel Limnology and Oceanography Research, Tel Shikmona, Haifa, Israel; 3 School of Environmental Sciences, University of East Anglia, Norwich, UK; 4 The Yigal Allon Kinneret Limnological Laboratory, Israel Oceanographic and Limnological Research, Migdal, Israel; 5 Quadram Institute Bioscience, Norwich Research Park, Norwich, UK; 6 Department of Marine Biology, Leon H. Charney School of Marine Sciences, University of Haifa, Haifa, Israel

**Keywords:** Lake sediment, Aerobic methanotrophy, Methanogenic zone, Hypoxia, *Methylomonas*, *Methylobacter*

## Abstract

**Background:**

Microbial methane oxidation, methanotrophy, plays a crucial role in mitigating the release of the potent greenhouse gas methane from aquatic systems. While aerobic methanotrophy is a well-established process in oxygen-rich environments, emerging evidence suggests their activity in hypoxic conditions. However, the adaptability of these methanotrophs to such environments has remained poorly understood. Here, we explored the genetic adaptability of aerobic methanotrophs to hypoxia in the methanogenic sediments of Lake Kinneret (LK). These LK methanogenic sediments, situated below the oxidic and sulfidic zones, were previously characterized by methane oxidation coupled with iron reduction via the involvement of aerobic methanotrophs.

**Results:**

In order to explore the adaptation of the methanotrophs to hypoxia, we conducted two experiments using LK sediments as inoculum: (i) an aerobic "classical" methanotrophic enrichment with ambient air employing DNA stable isotope probing (DNA-SIP) and (ii) hypoxic methanotrophic enrichment with repeated spiking of 1% oxygen. Analysis of 16S rRNA gene amplicons revealed the enrichment of *Methylococcales* methanotrophs, being up to a third of the enriched community. *Methylobacter*, *Methylogaea*, and *Methylomonas* were prominent in the aerobic experiment, while hypoxic conditions enriched primarily *Methylomonas*. Using metagenomics sequencing of DNA extracted from these experiments, we curated five *Methylococcales* metagenome-assembled genomes (MAGs) and evaluated the genetic basis for their survival in hypoxic environments. A comparative analysis with an additional 62 *Methylococcales* genomes from various environments highlighted several core genetic adaptations to hypoxia found in most examined *Methylococcales* genomes, including high-affinity cytochrome oxidases, oxygen-binding proteins, fermentation-based methane oxidation, motility, and glycogen use. We also found that some *Methylococcales*, including LK *Methylococcales,* may denitrify, while metals and humic substances may also serve as electron acceptors alternative to oxygen. Outer membrane multi-heme cytochromes and riboflavin were identified as potential mediators for the utilization of metals and humic material. These diverse mechanisms suggest the ability of methanotrophs to thrive in ecological niches previously thought inhospitable for their growth.

**Conclusions:**

Our study sheds light on the ability of enriched *Methylococcales* methanotrophs from methanogenic LK sediments to survive under hypoxia. Genomic analysis revealed a spectrum of genetic capabilities, potentially enabling these methanotrophs to function. The identified mechanisms, such as those enabling the use of alternative electron acceptors, expand our understanding of methanotroph resilience in diverse ecological settings. These findings contribute to the broader knowledge of microbial methane oxidation and have implications for understanding and potential contribution methanotrophs may have in mitigating methane emissions in various environmental conditions.

**Supplementary Information:**

The online version contains supplementary material available at 10.1186/s40793-024-00586-1.

## Background

Methane, a potent greenhouse gas, has more than threefold increased its atmospheric concentrations since the pre-industrial era. Around half of global methane emissions arise from natural inland waters production, including lake sediments, wetlands, rivers, and reservoirs [1]. Methane is consumed naturally by aerobic and anaerobic methanotrophs. Aerobic methanotrophy is observed in oxic environments and performed by aerobic bacterial methanotrophs, employing oxygen for methane activation and catalyze the oxidation of methane to methanol through the action of methane monooxygenase (MMO) [2, 3]. Anaerobic oxidation of methane (AOM) can be linked to sulfate reduction through anaerobic archaeal methanotrophs (ANMEs) and sulfate-reducing bacteria [4]. This process effectively prevents the release of up to 90% of the produced methane in marine sediments, and can be efficient also in rich sulfate freshwater systems [5]. In sulfate-depleted environments such as most freshwater sediments, AOM can consume over 50% of the produced methane [6] and be coupled to other electron acceptors such as nitrate, nitrite, metal oxides, and humic substances [7–11].

Microorganisms participating in AOM have been identified not only as ANMEs but surprisingly also as those considered aerobic bacterial methanotrophs. These bacteria were observed to be active and involved in methane oxidation beneath the chemocline in the anoxic hypolimnion [12, 13] and in certain freshwater lake sediments [14–17]. Certain methanotrophs employ unique mechanisms to obtain oxygen, such as the disproportionation of various molecules. For instance, *Methylomirabilis* (NC10) produces oxygen through the disproportionation of nitrite, utilizing it subsequently for the oxidation of methane [18]. Additionally, specific alphaproteobacterial methanotrophs utilize methanobactins to generate both oxygen and hydrogen through the disproportionation of water. [19]. Some bacterial methanotrophs exhibit versatility by utilizing alternative electron acceptors. *Methylococcales* bacteria *Methylomonas denitrificans* and *Methylocaldum* sp. have been experimentally shown to couple denitrification with methane oxidation [20–22], while others, such as *Methylomonas*, *Methylosinus* and *Methylococcus capsulatus* demonstrate methane oxidation coupled to iron reduction [23–25]. The adaptation mechanisms, that enable such a switch, remain poorly understood.

Here we explore the remarkable genetic adaptability of *Methylococcales* to hypoxia in methanogenic sediments of Lake Kinneret (LK, Sea of Galilee), where our previous studies confirmed methane oxidation coupled to iron reduction (Fe-AOM) beneath the sulfate reduction zone in the iron rich methanogenic zone [26]. The mediation of this Fe-AOM process was proposed to involve archaea methanogens and bacterial methanotrophs [27–29]. In-depth analyses, including isotopes of specific fatty acid lipids, quantification of the functional gene *pmoA*, and metagenomic analysis, confirmed the involvement of *Methylococcales*-like methanotrophs in methane oxidation [15, 27, 28]. Aerobic methanotrophy was also shown to boost iron reduction in these sediments [25], but the potential microbial mechanisms that may allow the methanotrophs to survive under hypoxia and stimulate iron reduction have remained unclear.

We hypothesized that LK methanogenic sediments harbor *Methylococcales* with unique strategies for survival in oxygen-limited conditions, and this research aimed to identify the key mechnisems enabling it. In order to explore this adaptation to hypoxia we conducted two experiments using LK methanogenic sediments as inoculum: (i) an aerobic "classic" methanotrophic enrichment with ambient air employing DNA stable isotope probing (DNA-SIP) and (ii) hypoxic methanotrophic enrichment with repeated spiking of 1% oxygen. Using metagenomics sequencing of DNA extracted from these experiments, we evaluated the genetic basis for their survival by comparing *Methylococcales* metagenome-assembled genomes (MAGs) from LK to *Methylococcales* genomes from diverse lineages and environments. including the. We investigated the genomic potential associated with various survival mechanisms including oxygen acquisition strategies, metabolic versatility and use of alternative electron acceptors**.**

## Methods

### Sampling site

LK is a monomictic lake in northern Israel, stratified usually between March and December, leading to anoxic hypolimnion [30]. The seasonal changes lead to variations in the geochemical porewater profiles in the sediments, primarily of methane, iron, and sulfur [26]. In the mixed period, the oxic-anoxic boundary reaches the sediment–water interface, affecting chemical profiles within the sediment. Sulfate in the sediment is depleted at around a depth of 10–20 cm depending on the stratification period and is followed by iron reduction. Methane concentration profiles generally increase with depth up to saturation levels, however, methane concentration profiles also hint at the presence of a "deep sink" correlated with an increase in concentrations of reduced iron [15, 26]. We collected sediment as inoculum for both experiments from the deepest point of LK (station A) using a gravity corer, as previously described by Bar-Or et al. [27]. The specific sediment used for the experiments originated from a depth of 25–40 cm below the sediment surface.

### Study design overview

**Fig. 1:**
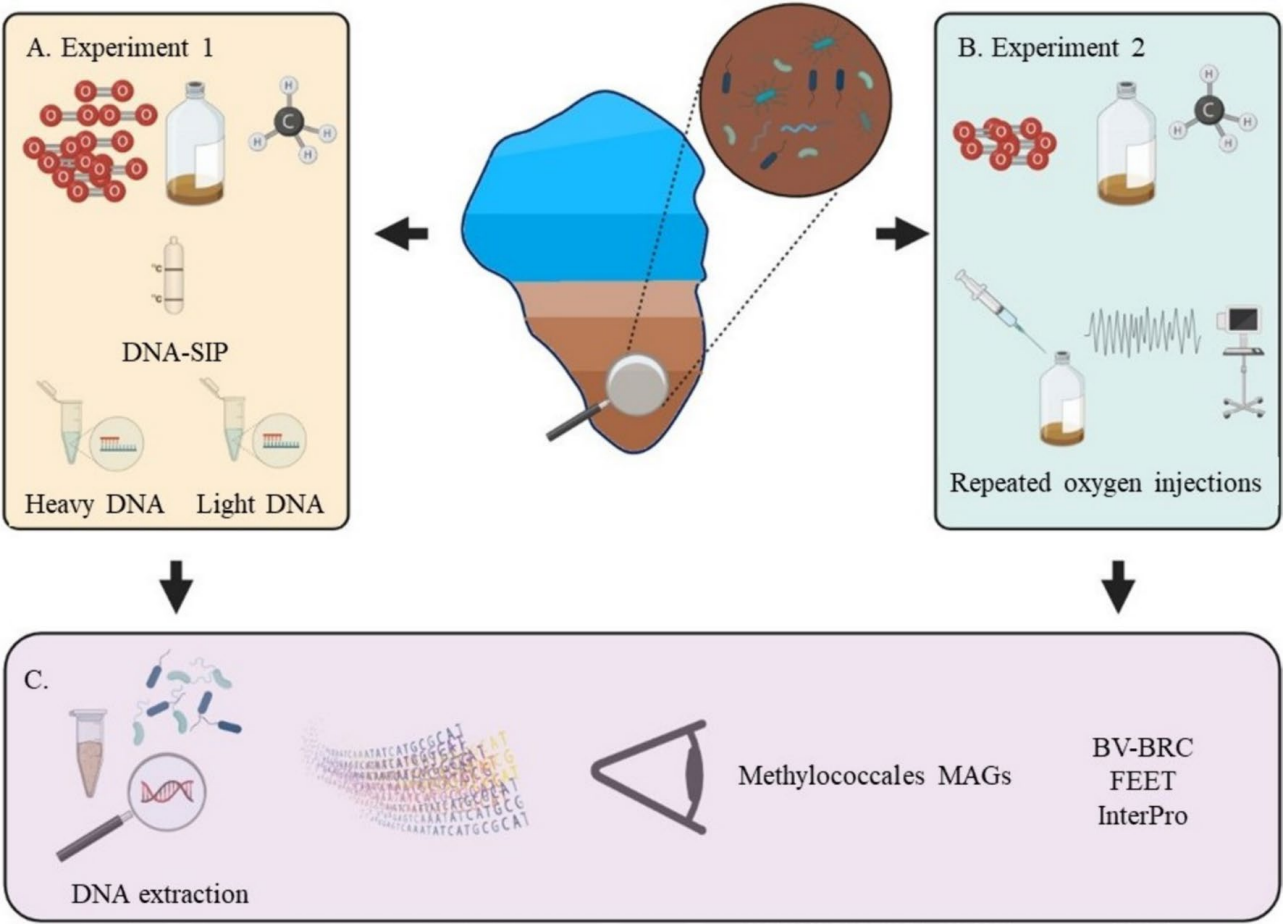
Experimental layout. Experimental design—sediment cores were collected from the methanotrophic zone in Lake Kineret and used to initiate two enrichment experiments. **A** Experiment 1: Incubated with ambient air, representing a "classical" enrichment approach coupled with DNA-SIP analysis. **B** Experiment 2: Hypoxic enrichment by repeatedly introducing small amounts (spikes) of oxygen (1%) to maintain low-oxygen conditions. Extracted DNA was used for 16S rRNA amplicon sequencing, and representing samples were also used to assemble and bin Methylococcales MAGs. Genome-based metabolic comparative analyses were performed using different bioinformatic platforms **C**

To achieve this study's aims, we designed and conducted two experiments intended mainly to enrich methanotrophs. Experiment 1 was a "classical" enrichment with ambient air, combined with DNA-Stable Isotope Probing (DNA-SIP). This experiment focused on identifying the active methanotrophs thriving under oxic conditions (Fig. 1A). In the second experiment, we stimulated methanotroph growth under hypoxic conditions (Fig. 1B). Following enrichments, we used amplicon sequencing of the 16S rRNA gene to study microbial diversity. Using metagenomic sequencing of representative samples, we curated *Methylococcales* MAGs. We investigated functions involved in adaptation to anoxia in these MAGs, alongside other *Methylococcales* genomes, using various bioinformatic platforms (Fig. 1C).

### Experiment 1—ambient air enrichment and DNA stable isotope fingerprinting

Sediment samples collected from LK in September 2019 were used to enrich methanotrophs and explore the active methane-utilizing organisms. Approximately 5 g of homogenized sediment was added to 300 ml sealed serum vials with black rubber septa. The vials were pre-filled with modified DNMS medium [31] (1:10 w/v). Prior to inoculation, the headspace of each vial was purged with nitrogen gas for a brief period (e.g., 1 h) to flush out any methane gas remaining in the sediment. Following this, the headspace was replaced with ambient air. Subsequently, either ^12^C-methane (control) or ^13^C-methane (used for DNA-SIP) was introduced at a concentration of 20% (v/v) as the sole carbon source. Incubations proceeded at 25 °C in the dark on a 120 rpm rotary shaker for a total of 21 days. Triplicate incubations were performed for each treatment, with detailed information on sampling times provided in the Supplementary Information (SI, Table S1). A separate 5 g sediment sample (time-zero control) was preserved in a 15 ml Falcon tube at − 20 °C for subsequent DNA extraction.

After incubation, sediment samples were carefully collected and processed for DNA extraction. Briefly, samples were centrifuged, supernatants discarded, and the resulting pellets were stored at − 20 °C. DNA was then extracted from 0.5 g aliquots of each sample (time-zero, ^12^C-methane, and ^13^C-methane) using a PowerSoil kit (Qiagen) following the manufacturer's instructions. To achieve sufficient DNA concentration for DNA-SIP analysis, seven aliquots from each sample were extracted and pooled. DNA quality and quantity were assessed using a NanoDrop Spectrophotometer (Thermo Fisher Scientific).

For DNA-SIP, samples were further processed through CsCl density gradient using a Beckman Coulter ultracentrifuge and a VTi 65.2 rotor, operating at 44,100 rpm at 20 °C for 65 h, as previously outlined [32]. Twelve fractions were collected from each sample, and their DNA content and their density was determined using an AR200 digital hand-held refractometer (Reichert, Buffalo, NY, USA). These DNA fractions were recovered, purified by PEG-6000 precipitation, resuspended in 30 μl TE buffer, and the DNA quantity was validated using Fluorometer (Qubit, Invitrogen).

#### Experiment 2—hypoxic enrichments

This experiment attempted to enrich methanotrophs in LK sediments with low oxygen conditions following the methods of Vigderovich et al. [25]. Sediment samples collected in March 2021 set as the starting material for the experiment. Each vial contained 7g of methanogenic sediments from LK, 50 mg of hematite (10 mM final concentration) and anoxic filtered porewater (1:3 w/v), previously extracted from the same depth in a 60 ml serum vials sealed with black rubber septa. Hematite, an iron oxide mineral, can potentially serve as an alternative electron acceptor for methanotrophs under anoxic conditions [25]. The anoxic porewater further ensured the initial absence of oxygen within the vials. To establish these anoxic conditions, each vial underwent continuous nitrogen purging for about an hour. Following this, the vials were stored in the dark at 4 °C for 5 days. After the anoxic period, the enrichment process was initiated. Vials were vigorously mixed (vortexed) and flushed again with nitrogen for 15 min to ensure the removal of any residual oxygen. Subsequently, 1.5 ml of air was introduced into each vial, resulting in a final headspace oxygen concentration of 1% (v/v). Two treatment groups were established CH_4_ + O_2_ treatment and N_2_ + O_2_ as Control. For the CH_4_ + O_2_ treatment, eleven vials received an additional 1.5 ml of 99.95% methane gas, resulting in a final headspace oxygen concentration of 5% (v/v) allowing methanotrophs to potentially uptake methane as an energy source. For the N_2_ + O_2_ Control, triplicate vials were supplemented with an additional 1.5 ml of 99.999% nitrogen gas instead of methane, resulting in a final headspace oxygen concentration of 5% (v/v), serving as a control for the effect of methane availability. The enrichment incubations proceeded at 25 °C in the dark with the vials inverted to minimize headspace gas exchange. Oxygen consumption within the vials was monitored throughout the experiment using PSt6 sensors in designated vials. Vials were sacrificed at various time intervals, detailed in the Supplementary Information (Table S2) and the experiment lasted for 40 days. These samples were promptly stored at -80 °C for subsequent DNA/RNA extraction. DNA was successfully extracted from all samples using a PowerSoil kit (Qiagen) following the manufacturer's instructions. However, RNA concentrations using Quant-ittTM RiboGreen RNA kit (ThermoFisher) were found to be below the detection limit.

#### DNA library preparation and sequencing

A total of thirty-seven samples underwent 16S rRNA gene sequencing, while three samples were also allocated for metagenome analysis. Comprehensive details regarding the samples utilized in the DNA-SIP and hypoxic experiments can be found in Tables S1 and S2, respectively. Sequencing of the V4 region of the 16S rRNA gene was executed using the primer pair 515f–806r [33, 34], and the procedure was conducted via Illumina sequencing at Hylabs, Rehovot, Israel.

Metagenomic libraries were generated from three distinct DNA samples: one originating from the DNA-SIP experiment and two from the hypoxic experiment (refer to Tables S1 and S2 for detailed descriptions, respectively). Subsequently, the libraries underwent sequencing at Novogene, Singapore, with each sample producing 65–80 million 2 × 150 bp paired-end reads using Illumina NovaSeq. The library construction process employed the NEBNext® Ultra™ II DNA Library Prep Kit. [33, 34]

### Bioinformatics

For 16S rRNA gene amplicons, demultiplexed paired-end reads were analyzed within QIIME2 V2020.6 pipeline [35]. By applying the DADA2 pipeline [36], implemented in QIIME2, reads were truncated according to their quality plots, chimeras were removed, and reads were merged and grouped into amplicon sequence variants (ASVs). Taxonomy was assigned to ASVs by Silva 138 99% classifier [37]. Beta diversity was visualized by Principal Coordinate Analysis (PCoA), in which the dissimilarity between samples was estimated with a Bray–Curtis distance matrix using the distance and ordination functions in the R package phyloseq [38].

Metagenomes were assembled using SPAdes V3.15 with–meta k = 21,33,66,99,127 parameters [39], following adapter trimming and error correction with tadpole.sh, using the BBtools suite following read preparation with the BBtools suite (Bushnell, B, sourceforge.net/projects/bbmap/). Downstream mapping and binning of metagenome-assembled genomes (MAGs) were performed using DAStool, Vamb, Maxbin 2.0, and Metabat2 [40–43] within the Atlas V2.9.1 pipeline [44], using the genome dereplication nucleotide identity threshold of 0.975. MAG quality was verified using Checkm2 [45] and QUAST [46]. Functional annotation was carried out using the SEED implemented in Viral Bioinformatics Resource Center (BV-BRC) server [47], and key annotations were verified by BLASTing against the NCBI database.

Additional *Methylococcales* genomes, a total of 62 genomes (a comprehensive list can be found in Table S3), were generated based on BLASTp against LK predicted membrane-bound particulate methane monooxygenase protein, using the BV-BRC platform [48–50] (max hit: 20, Evalue threshold: 0.0001). The average nucleotide identity (ANI) was calculated using pyani [51] (See Table S4 for their values).

Multiheme cytochromes (MHCs) were assigned using the FEET pipeline (https://github.com/ McMahonLab/FEET.git) [52]. In short, FEET first uses python to find MHCs using the following regular expressions for heme binding motifs: [CXCH], [CXXCH], [CXXXCH], and [CXXXXXXXXXXX[! = C]XXCH]. At least three [CXXCH] motifs and at least five total aforementioned motifs were required to call an MHC. Localization was predicted by Cello V2.5 (http://cello.life.nctu.edu.tw/). Each protein sequence was then manually verified using the InterPro database (https://www.ebi.ac.uk/ InterPro/) [53].

Phylogenetic trees were generated with the BV-BRC platform [53]. Marker proteins that are universally conserved across the bacterial domain were extracted from genomes [54]. A hundred single-copy markers that were present in all genomes (See Table S5 for gene list) analyzed in this study were used for alignment with MUSCLE [55]. The randomized accelerated maximum likelihood (RAxML version 8.2.11) tree was calculated [56]. Final representation of the tree was curated using Itol version 6.9 (https://itol.embl.de/).

## Results and discussion

### Ambient oxygen selects for the enrichment of aerobic methanotrophs

**Fig. 2:**
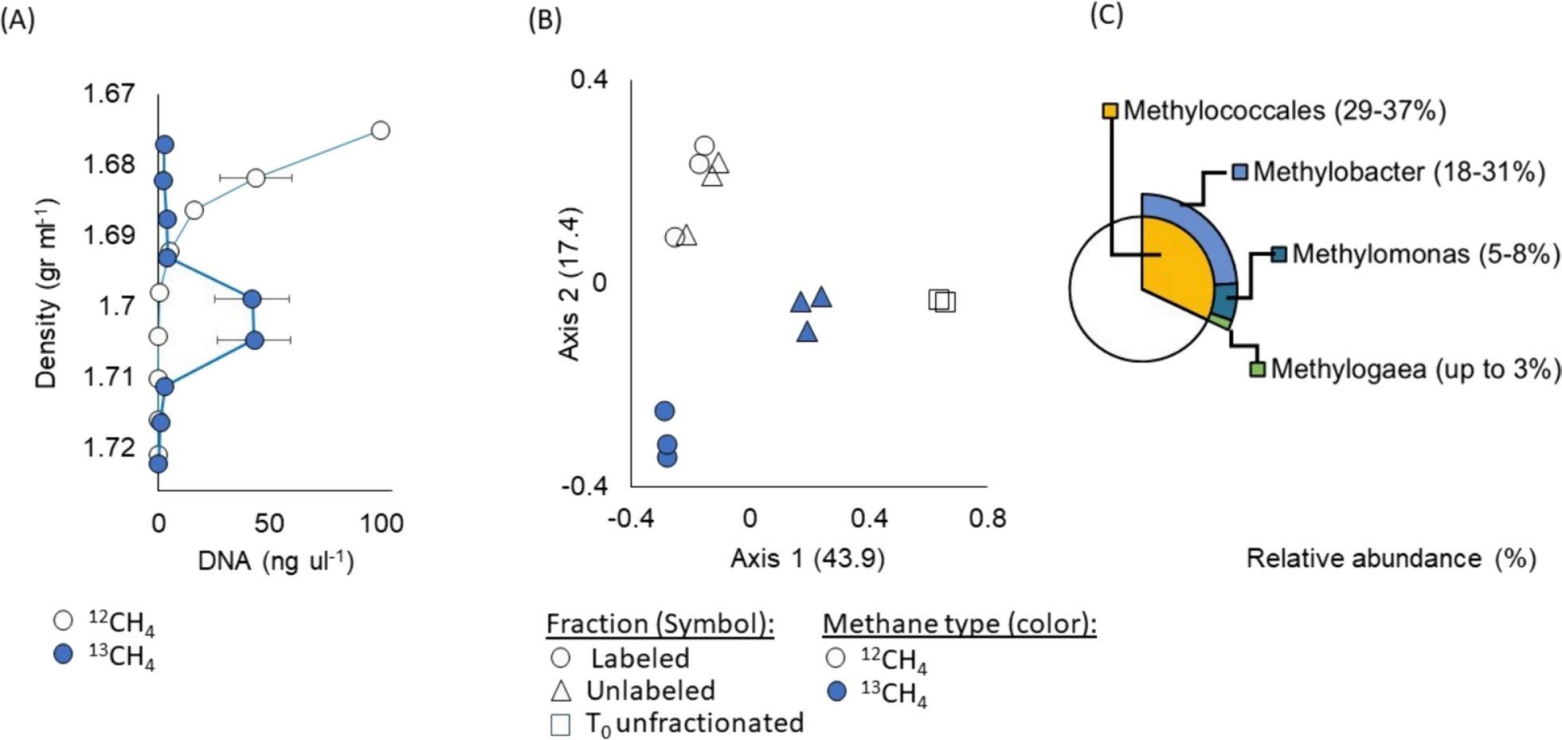
Aerobic enrichment experiment employing DNA-SIP. **A** The average DNA concentration (ng/μl) for each fraction (n = 3 biological replicates) in the ^13^C-methane-fed cultures (blue) and the ^12^C-methane-fed cultures (colorless). Error bars indicate the standard error (standard deviation/n) of the DNA concentration, while the error bars for density are smaller than the symbol. **B** Principal Component Analysis (PCoA) based on Bray–Curtis dissimilarity illustrating microbial diversity in labeled DNA fractions (circles), unlabeled DNA fractions (triangles), and time zero samples that were not fractionated (squares) for both ^13^C-methane-fed cultures (in blue) and ^12^C-methane-fed cultures (colorless). **C** The average relative abundance of dominant *Methylococcales* in the labeled DNA fractions of the ^13^C-methane-fed cultures

DNA-SIP experiments utilizing labeled ^13^C-methane resulted in the isolation of ^13^C-enriched DNA observed in fractions with a density ranging from 1.72 to 1.70 g ml^−1^, as illustrated in the labeled fraction (Fig. 2a). Analysis of the relative abundance of 16S rRNA genes revealed bacterial communities that differed between the labeled and unlabeled fractions, as evidenced by Principal Coordinates Analysis (PCoA) based on Bray–Curtis dissimilarity (Fig. 2b). Axis 1 and 2 explained 43.9% and 17.4% of the variation, respectively, indicating distinct communities in the labeled and unlabeled fractions, while ^12^C-methane-fed communities clustered together. The use of the ^12^C-methane-fed sample as a control accounted for differences in the guanine/cytosine (GC) content of DNA samples.

Despite *Methylococcales* comprising less than 1% of the initial microbial population at time zero, enrichment during the experiment resulted in *Methylococcales* accounting for approximately one-third of the microbial community (refer to Figure S1). In the fractions fed with labeled ^13^C-methane, *Methylobacter* emerged as the predominant taxon with a relative read abundance ranging from 18 to 31%, alongside the enrichment of other methanotrophs (Fig. 2c). This finding aligns with previous studies that identified *Methylobacter* as a dominant and active species in diverse natural environments [57], including oxic [58] and anoxic freshwater lake sediments [59], anoxic water columns [60], and wetlands [61]. Other enriched *Methylococcales* species in this experiment included *Methylomonas* (5–8%) and *Methylogaea* (up to 3%) (Fig. 2c). In contrast, within the ^12^C-methane-enriched cultures, *Methylobacter* exhibited a relative abundance of 6–10%, while the relative abundance of *Methylomonas* and *Methylogaea* varied significantly among biological triplicates, ranging from 4% to up to 20% (refer to Figure S1).

### Hypoxic conditions select for *Methylomonas*

**Fig. 3:**
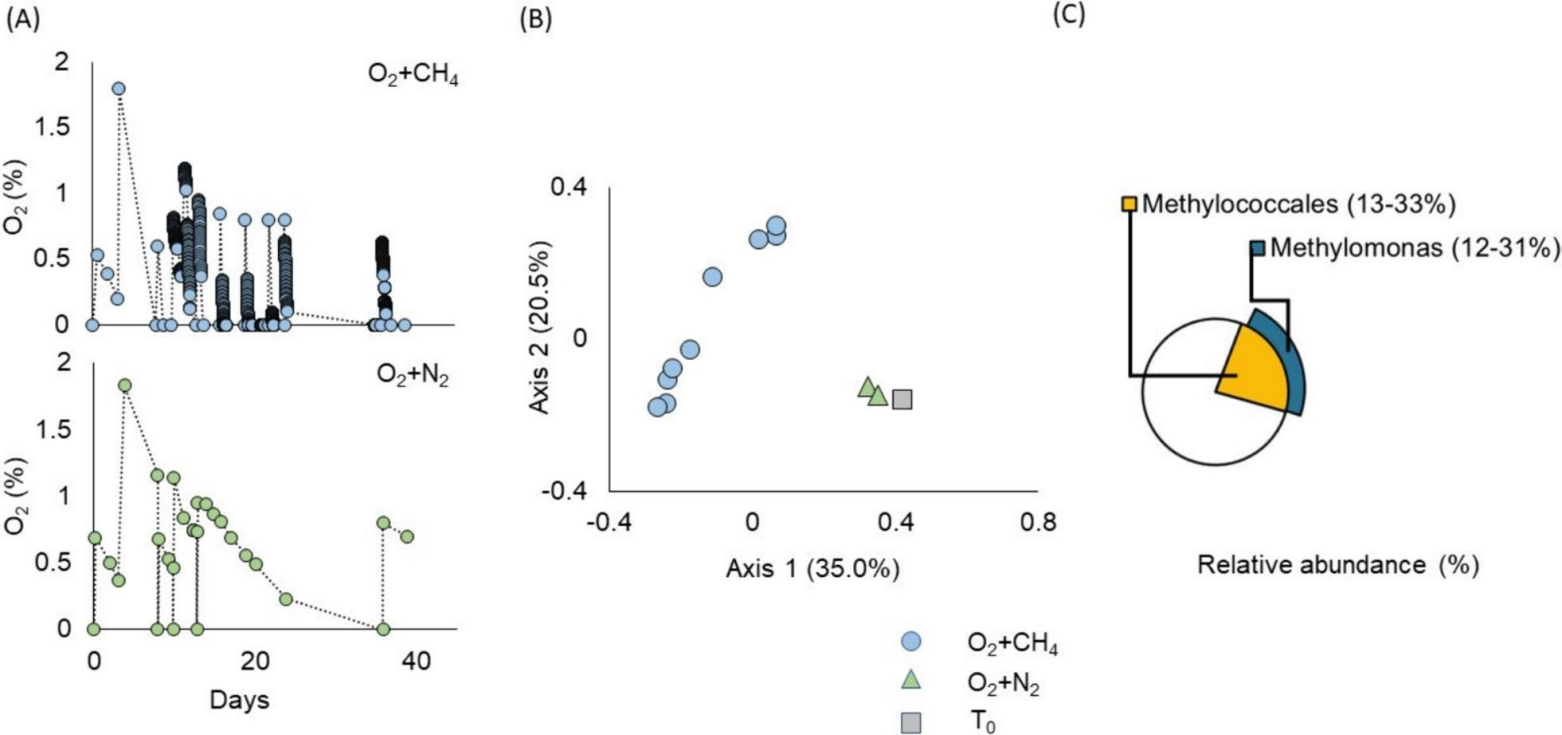
Hypoxic enrichment experiment. **A** In vitro monitoring of oxygen levels (%) in bottles exposed to O_2_ + CH_4_ (blue) and O_2_ + N_2_ (green). **B** Principal Component Analysis illustrating microbial diversity exposed to O_2_ + CH_4_ (circles), O_2_ + N_2_ (triangles), and time zero samples without enrichment (squares). These are color-coded based on treatment, with blue, green, and gray representing O_2_ + CH_4_, O_2_ + N_2_, and time zero, respectively. **C** The average relative abundance of dominant *Methylococcales* spp. enriched in the O_2_ + CH_4_ treatment

We observed a notable adaptation of microbial communities to periodic spiking with 1% oxygen and methane, leading to a significant increase in oxygen consumption (0.69 ± 0.04 mg L^−1^ h^−1^), in contrast to controls where the oxygen consumption was markedly lower (0.02 ± 0.01 mg L^−1^ h^−1^) (Fig. 3a). Microbial communities in cultures deprived of methane exhibited clustering with those from non-enriched samples (time zero), indicating that the microbial community in these samples remained relatively consistent despite exposure to oxygen. These findings suggest that methane serves as the electron donor for oxygen respiration, especially considering the limited availability of alternative electron donors in these methanogenic sediments (Fig. 3a).

Further analysis of the microbial community revealed the specific selection of microorganisms through periodic spiking with 1% oxygen (Fig. 3b), predominantly enriching for *Methylomonas* (Fig. 3c).

These outcomes align with previous studies, reinforcing the notion that *Methylomonas* may exhibit better adaptation to low oxygen conditions, such as, better uptake of oxygen and methane, compared to *Methylobacter* [62].

#### *Methylococcales* lineages dominate enrichment cultures

**Fig. 4:**
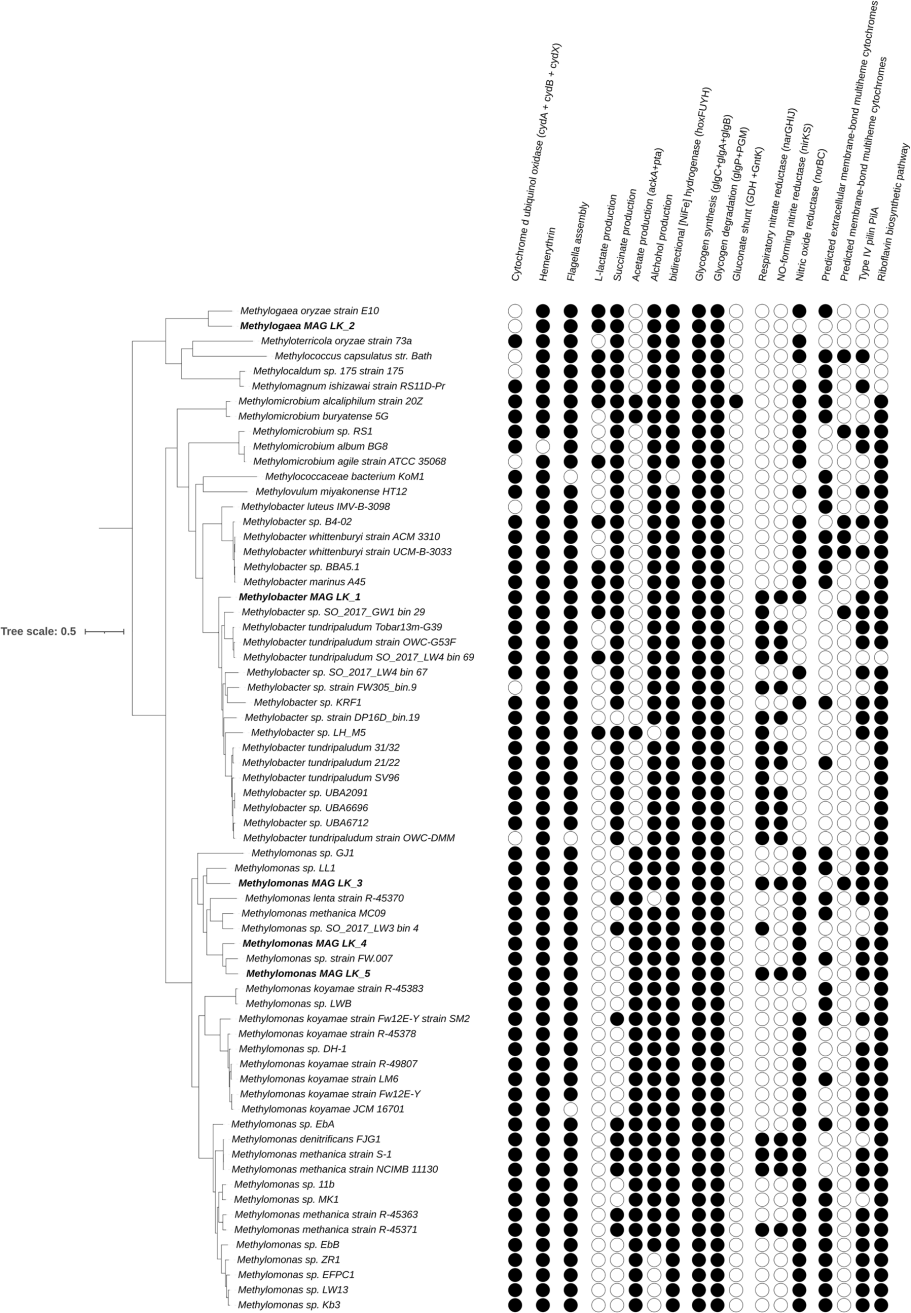
Phylogenomic analysis and metabolic profiling. A phylogenomic tree along with the metabolic presence-absence profile of 67 *Methylococcales* genomes. A comprehensive list of the proteins is available in Table S7 and predicted OMC proteins are available in Table S8. Additional information regarding presence-absence of kye genes particulate methane monooxygenase *pmoCAB* operon and *pxmABC* operon, soluble methane monooxygenase (mmoXYBZDC), lanthanide-dependent methanol dehydrogenases (xoxF), and methanol dehydrogenase (mxaF) is available in Table S9. Notably, the five LK *Methylococcales* are highlighted in bold. The phylogenetic tree is built on 100 genes (refer to Table S5), and the taxa clustering percentage is based on 100 bootstrap resamples, consistently yielding values of 98 or higher (specific values not shown)

In this investigation, methanotrophs were selectively enriched from methanogenic LK sediments through two distinct enrichment experiments. The first experiment involved the use of ambient air coupled with DNA-stable isotope probing (DNA-SIP), while the second experiment included repeated injections of 1% oxygen to simulate hypoxic conditions. From both experiments, we identified five *Methylococcales* metagenome-assembled genomes (MAGs), detailed in Table 1 for genome statistics. The metagenomic analysis uncovered the enrichment of *Methylomonas, Methylogaea,* and *Methylobacter* (MAGs 1–5 respectively). The closest relatives to these lineages were *Methylomonas* sp. ZR1 [63], *Methylogaea oryzae* strain E10 [64] and *Methylobacter tundripaludum* strain OWC-G53F [63] (see Fig. 4 and Table 2 for ANI values). For the hypoxic enrichments, two MAGs, namely, *Methylomonas* LK_4 and LK_5, which are related to *Methylomonas* sp. strain FW.007 [65]. A detailed genomic comparison between all LK *Methylococcales* MAGs highlighting functions of interest is given below and summarized in Table 2.

**Table 1:** Genome assembly statistics

Originally enriched in experiment	MAG	Genome Name	Size (mb^a^)	Contigs N50 (mb^a^)	CheckM Completeness (%)	CheckM Contamination (%)	Relative abundance (%)	Closest ANI^b^	ANI^b^ (%)
DNA-SIP	LK_1	*Methylobacter*	3.96	0.10	99.2	1.63	40.6	*Methylobacter tundripaludum* strain OWC-G53F* (GCA_002934365.1)*	84
	LK_2	*Methylogaea*	3.90	0.03	98.2	4.95	2.1	*Methylogaea oryzae* strain E10* (GCA_019669985.1)*	81
	LK_3	*Methylomonas*	4.15	0.08	99.3	0.46	6.9	*Methylomonas* sp. ZR1 (*GCA_013141865.1)*	76
Hypoxic	LK_4	*Methylomonas*	4.04	0.28	99.9	0.17	35.1	*Methylomonas* sp. strain FW.007* (GCA_002928965.1)*	84
	LK_5	*Methylomonas*	4.76	0.08	99.9	0.69	6.5		84

^a^mb—million base pairs, ^b^ANI—Average nucleotide identity

**Table 2:** List of presence/ absence functions

Originally enriched in exp		*Methylobacter* LK_1	*Methylomonas* LK_2	*Methylogaea* LK_3	*Methylomonas* LK_4	*Methylomonas* LK_5
		A			B	
C1	*pmoCAB* operon	+	+	−	+	+
	*pmoABC* operon	−	−	+	+	+
	*mxaF*	-	+	−	+	+
	*xoxF*	+	+	−	+	+
	RuMP	+	+	+	+	+
	ED	+	+	−	+	+
	EMP	+	+	+	+	+
	Serine cycle	−	−	−	−	−
	TCA	+	+	−	+	+

Detailed description of all predicted proteins used to construct this table are listed in Table S6

### Metabolic reconstruction of novel Lake Kinneret methanotrophs

Genome-base metabolic reconstruction of the five MAGs affirmed the characteristic metabolic framework of *Methylococcales* methanotrophs (Table 2). The prediction of methane oxidation through the particulate methane monooxygenase was consistent across all LK *Methylococcales* lineages. With the exception of *Methylogaea* LK_2, which exclusively harbored the *pxmABC* genes encoding an alternative form of particulate methane monooxygenase potentially functioning under oxygen-limiting conditions [20], the remaining LK *Methylococcales* lineages were predicted to possess one copy of the *pmoCAB* operon, encoding the canonical particulate methane monooxygenase. The genomes of Methylomonas LK_4 and LK_5 contained both *pxmABC* and *pmoCAB* operons, potentially broadening their range of affinities for methane and oxygen [66]. In addition, none of the genomes of the LK *Methylococcales* lineages encoded the soluble methane oxygenase.

The lanthanide-dependent *xoxF* [67] was predicted to catalyze methanol oxidation in all LK *Methylococcales* lineages except *Methylogaea*, whereas the calcium-dependent *mxaFI* [68] was predicted only in the genome of LK *Methylomonas* and likely within the *Methylogaea* LK_2 genome (only mxaI was found). The potential secretion of methanol by methanotrophs into the environment, with subsequent consumption by syntrophic partners, indicative of cross-feeding between methanotrophs and methylotrophs [69], was considered highly likely. This inference is supported by the elevated relative abundance of *Methylotenera* in our enrichment experiments (refer to Figures S1 and S2 for microbial community relative abundance).

The presence of the ribulose monophosphate pathway (RuMP) was identified in all LK *Methylococcales* lineages, evident through the presence of key genes such as those that encode the 3-hexulose-6-phosphate synthase (*hps*) and 6-*phospho*-3-hexuloisomerase (*phi*) [70]. The RuMP pathway appears to be the exclusive pathway for methane carbon assimilation, as the serine cycle was absent, lacking genes encoding key enzymes like malate thiokinase (both *mtkAB*) and hydroxypyruvate reductase (*ghrB*). Except for *Methylogaea*, all LK *Methylococcales* lineages encoded the phosphogluconate dehydratase (*edd)* and 2-dehydro-3-deoxy-phosphogluconate aldolase* (eda)* genes necessary for the Entner–Doudoroff (ED) variant of the RuMP cycle [71]. The energy-efficient Embden–Meyerhof–Parnas (EMP) variant [71] was predicted in all *Methylococcales* lineages, evidenced by the presence of non-ATP dependent pyrophosphate-dependent phosphofructokinase (*pfk*), triosephosphate isomerase (*tpi*), glyceraldehyde 3-phosphate dehydrogenase (*gapdh*), phosphoglycerate kinase (*pgk*), and enolase (*eno*) genes.

### Adaptations to hypoxic conditions in *Methylococcales*

To assess whether the genomic adaptations to hypoxic conditions are unique to LK *Methylococcales*, we compared their genomes and with an additional 62 *Methylococcales* genomes from diverse environments (Fig. 4). These genomes represent *Methylococcales* from groundwater, contaminated rivers, sewage systems, lake sediments, rice fields, volcano mud, and others (Supplementary Table S3).

This comparison revealed a core set of mechanisms shared by most *Methylococcales*, including those from LK potentially enabling them to function effectively in hypoxic conditions. These include (i) Enhanced oxygen usage by cytochrome bd ubiquinol oxidase (found in 88% of analyzed genomes), allowing efficient oxygen respiration potentially sustaining growth at ≤ 3 nM molecular oxygen [72]. (ii) Enhancing respiration under hypoxia by oxygen-binding hemerythrin, by increasing the activity of pMMO [73] (present all of the analyzed genomes). (iii) Flagella-mediated motility across gradients to optimize oxygen and methane availability (present in 95% of the analyzed genomes) [74]. (iv) Alternative electron acceptors including nitric oxide reduction, as well as riboflavins (found in 70% and 89% of the genomes, respectively). These last electron acceptors, are soluble secreted electron shuttles mediating extracellular electron transfer (EET) [75–77], which can be reversibly oxidized and reduced, carrying electrons between cells and insoluble electron acceptors such as manganese and iron oxides over large distances [71, 75, 78]. Manganese and iron oxides are highly abundant in methanogenic sediments [79].

*Methylococcales* can store carbohydrates and use alternative metabolic pathways to provide energy under oxygen limitation. Genes for glycogen synthesis and degradation were found in all of the genomes, likely allowing *Methylococcales* to conserve resources during periods of limited nutrients [80, 81]. Additional metabolic alternative is the ability to produce fermentation products like succinate and acetate was found in 66% and 51% of genomes, respectively. The occurrence of alcohol dehydrogenases (present in 91% of genomes) and bidirectional hydrogenases (present in 99% of genomes) indicates potential for alcohol and hydrogen production [60, 82].

While many metabolic mechanisms were shared among *Methylococcales*, we identified several less abundant mechanisms that were present in LK *Methylococcales*. These include (i) production of lactate (found in 21% of the genomes), predicted in *Methylobacter* LK_1 and *Methylogaea* LK_2 MAGs; (ii) Methane-dependent denitrification serves as a link between the carbon and nitrogen cycles [20, 83]. While nitrous oxide reduction was found as a common trait, respiratory nitrate reductase and nitric oxide-forming nitrite reductase (with respective genes identified in 36% and 27% of the genomes) were observed as less prevalent mechanisms. However, despite being relatively uncommon among the *Methylococcales*, these mechanisms were found more abundant in LK *Methylococcales* and exhibited in Methylobacter LK_1, Methylomonas LK_3, and LK_5 MAGs; (iii) Outer membrane cytochromes (OMCs, found in 62% of the genomes) needed to reduce iron [24, 59, 84], were found in *Methylomonas* LK_3, LK_4 and LK_5. *Methylococcales* appear to lack electrically conductive pili (e-pili) that can support EET [84], as > 120 amino acid long PilA proteins in *Methylococcales* were longer than the canonical 60–90 amino acid-long e-pili [85].

Additional manual investigation of OMCs using the InterPro database confirmed that at least some of these sequences belong to the multiheme cytochrome superfamily with at least one copy of predicted as extracellular OMC (found in 55% of the genomes), and fewer OMCs were predicted as membrane-bound (found in 10% of the genomes). Others were either unrelated to OMCs or predicted to be hydroxylamine oxidoreductase or cytochromes c-552 involved in ammonia oxidation and nitrite reduction. Among LK *Methylococcales*, only *Methylomonas* LK_3 encoded a membrane-associated OMC, hinting at the possibility of EET in this organism, but this strategy to cope with oxygen limitation is not widespread in LK.

## Conclusions

Our study highlights the potential prevalence and diversity of adaptive strategies utilized by methanotrophic bacteria in low-oxygen environments, specifically within LK sediments. These LK sediments, located 20 cm below the sediment water interface and 20 m below the hypolimnion, were previously shown to be involve *Methylococcales* in methane oxidation and stimulation of iron reduction. We hypothesized that this environment harbors *Methylococcales* with unique strategies for survival. Through enrichment experiments, comparative metagenomics, and genomic analyses of diverse *Methylococcales* lineages, we propose several potential mechanisms enabling these organisms to thrive under oxygen-limited conditions. Our findings demonstrate that most *Methylococcales*, including those from LK, possess a set of traits enabling their survival in hypoxic environments: spanning effective usage of trace oxygen, motility for reaching optimal oxygen concentrations, glycogen storage, alternative energy generation using fermentation, and the use of alternative electron acceptors and possible EET mediated by riboflavins. Some less widespread functions employed by LK *Methylococcales* include lactate productionn, methane-dependent denitrification, and EET via outer membrane cytochromes (OMCs). Further studies are necessary to validate these potential strategies. Experimental examination of these potential adaptations may unveil additional layers of complexity in the survival strategies. Additionally, investigating whether these adaptations are expressed under hypoxic conditions is essential for understanding their role in hypoxia tolerance. This knowledge may contribute to a more comprehensive understanding of global methane cycling.

## Supplementary Information

Additional file 1.

Additional file 2.

## Data Availability

The datasets generated and analyzed during the current study are available under NCBI BioProject ID PRJNA1041615. Additional data are available under the Supplementary information sections.

## References

[1] Johnson MS, Matthews E, Du J, Genovese V, Bastviken D. Methane emission from global lakes: new spatiotemporal data and observation-driven modeling of methane dynamics indicates lower emissions. JGR Biogeosci. 2022;127(7):e2022JG006793. 10.1029/2022JG006793 PMC9540782 36250198

[2] Kelly DP, Wood AP. The chemolithotrophic prokaryotes. Prokaryotes. 2006. 10.1007/0-387-30742-7_15. 10.1007/0-387-30742-7_15

[3] Lawton TJ, Rosenzweig AC. Biocatalysts for methane conversion: big progress on breaking a small substrate. Curr Opin Chem Biol. 2016;35:142–9. 27768948 10.1016/j.cbpa.2016.10.001 PMC5161620

[4] Knittel K, Boetius A. Anaerobic oxidation of methane: progress with an unknown process. Annu Rev Microbiol. 2009;63:311–34. 19575572 10.1146/annurev.micro.61.080706.093130

[5] Nordi KÁ, Thamdrup B, Schubert CJ. Anaerobic oxidation of methane in an iron-rich Danish freshwater lake sediment. Limnol Oceanogr. 2013;58:546–54. 10.4319/lo.2013.58.2.0546

[6] Segarra KEA, et al. High rates of anaerobic methane oxidation in freshwater wetlands reduce potential atmospheric methane emissions. Nat Commun. 2015;6:2–9. 10.1038/ncomms8477 26123199

[7] Haroon MF, et al. Anaerobic oxidation of methane coupled to nitrate reduction in a novel archaeal lineage. Nature. 2013;500:567–70. 23892779 10.1038/nature12375

[8] Raghoebarsing AA, et al. A microbial consortium couples anaerobic methane oxidation to denitrification. Nature. 2006;440:918–21. 16612380 10.1038/nature04617

[9] Nordi K, Thamdrup B. Nitrate-dependent anaerobic methane oxidation in a freshwater sediment. Geochim Cosmochim Acta. 2014;132:141–50. 10.1016/j.gca.2014.01.032

[10] Ettwig KF, et al. Archaea catalyze iron-dependent anaerobic oxidation of methane. Proc Natl Acad Sci U S A. 2016;113:12792–6. 27791118 10.1073/pnas.1609534113 PMC5111651

[11] Lu YZ, et al. Cr(VI) reduction coupled with anaerobic oxidation of methane in a laboratory reactor. Water Res. 2016;102:445–52. 27395029 10.1016/j.watres.2016.06.065

[12] Blees J, et al. Micro-aerobic bacterial methane oxidation in the chemocline and anoxic water column of deep south-Alpine Lake Lugano (Switzerland). Limnol Oceanogr. 2014;59:311–24. 10.4319/lo.2014.59.2.0311

[13] Oswald K, et al. Aerobic gammaproteobacterial methanotrophs mitigate methane emissions from oxic and anoxic lake waters. Limnol Oceanogr. 2016;61:S101–18. 10.1002/lno.10312

[14] Beck DAC, et al. A metagenomic insight into freshwater methane-utilizing communities and evidence for cooperation between the *Methylococcaceae* and the *Methylophilaceae*. PeerJ. 2013;2013:1–23. 10.7717/peerj.23 PMC3628875 23638358

[15] Bar-Or I, Ben-Dov E, Kushmaro A, Eckert W, Sivan O. Methane-related changes in prokaryotes along geochemical profiles in sediments of Lake Kinneret (Israel). Biogeosciences. 2015;12:2847–60. 10.5194/bg-12-2847-2015

[16] Martinez-Cruz K, et al. Anaerobic oxidation of methane by aerobic methanotrophs in sub-Arctic lake sediments. Sci Total Environ. 2017;607–608:23–31. 28686892 10.1016/j.scitotenv.2017.06.187

[17] Su G, Zopfi J, Niemann H, Lehmann MF. Multiple groups of methanotrophic bacteria mediate methane oxidation in anoxic lake sediments. Front Microbiol. 2022;13:864630. 35615497 10.3389/fmicb.2022.864630 PMC9125203

[18] Ettwig KF, et al. Nitrite-driven anaerobic methane oxidation by oxygenic bacteria. Nature. 2010;464:543–8. 20336137 10.1038/nature08883

[19] Dershwitz P, et al. Oxygen generation via water splitting by a novel biogenic metal ion-binding compound. Appl Environ Microbiol. 2021;87:1–14. 10.1128/AEM.00286-21 PMC8231713 33962982

[20] Kits KD, Klotz MG, Stein LY. Methane oxidation coupled to nitrate reduction under hypoxia by the Gammaproteobacterium *Methylomonas denitrificans* sp. Nov. type strain FJG1. Environ Microbiol. 2015;17:3219–32. 25580993 10.1111/1462-2920.12772

[21] Orata FD, Kits KD, Stein LY. Complete genome sequence of *Methylomonas denitrificans* strain FJG1, an obligate aerobic methanotroph that can couple methane oxidation with denitrification. Genome Announc. 2018;6:1–2. 10.1128/genomeA.00276-18 PMC5920175 29700144

[22] Dang CC, et al. Heavy metal reduction coupled to methane oxidation: mechanisms, recent advances and future perspectives. J Hazard Mater. 2021;405:124076. 33268204 10.1016/j.jhazmat.2020.124076

[23] Zheng Y, et al. Methane-dependent mineral reduction by aerobic methanotrophs under hypoxia. Environ Sci Technol Lett. 2020. 10.1021/acs.estlett.0c00436.33195731 10.1021/acs.estlett.0c00436

[24] Tanaka K, et al. Extracellular electron transfer via outer membrane cytochromes in a methanotrophic bacterium *Methylococcus capsulatus* (Bath). Front Microbiol. 2018;9:1–7. 30555443 10.3389/fmicb.2018.02905 PMC6281684

[25] Vigderovich H, et al. Aerobic methanotrophy increases the net iron reduction in methanogenic lake sediments. Front Microbiol. 2023;14:1–17. 10.3389/fmicb.2023.1206414 PMC10415106 37577416

[26] Sivan O, et al. Geochemical evidence for iron-mediated anaerobic oxidation of methane. Limnol Oceanogr. 2011;56:1536–44. 10.4319/lo.2011.56.4.1536

[27] Bar-Or I, et al. Iron-coupled anaerobic oxidation of methane performed by a mixed bacterial-archaeal community based on poorly reactive minerals. Environ Sci Technol. 2017;51:12293–301. 28965392 10.1021/acs.est.7b03126

[28] Elul M, et al. Metagenomic insights into the metabolism of microbial communities that mediate iron and methane cycling in Lake Kinneret sediments. 2020. Biogeosci Discuss. 10.5194/bg-2020-329. 10.5194/bg-2020-329

[29] Vigderovich H, et al. Long-term incubations provide insight into the mechanisms of anaerobic oxidation of methane in methanogenic lake sediments. Biogeosciences. 2022;19:2313–31. 10.5194/bg-19-2313-2022

[30] Adler M, Eckert W, Sivan O. Quantifying rates of methanogenesis and methanotrophy in Lake Kinneret sediments (Israel) using pore-water profiles. Limnol Oceanogr. 2011;56:1525–35. 10.4319/lo.2011.56.4.1525

[31] Farhan Ul Haque M, Crombie AT, Murrell JC. Novel facultative Methylocella strains are active methane consumers at terrestrial natural gas seeps. Microbiome. 2019;7:1–17. 31585550 10.1186/s40168-019-0741-3 PMC6778391

[32] Neufeld JD, et al. DNA stable-isotope probing. Nat Protoc. 2007;2:860–6. 17446886 10.1038/nprot.2007.109

[33] Parada AE, Needham DM, Fuhrman JA. Every base matters: assessing small subunit rRNA primers for marine microbiomes with mock communities, time series and global field samples. Environ Microbiol. 2016;18:1403–14. 26271760 10.1111/1462-2920.13023

[34] Apprill A, Mcnally S, Parsons R, Weber L. Minor revision to V4 region SSU rRNA 806R gene primer greatly increases detection of SAR11 bacterioplankton. Aquat Microb Ecol. 2015;75:129–37. 10.3354/ame01753

[35] Bolyen E, et al. Reproducible, interactive, scalable and extensible microbiome data science using QIIME 2. Nat Biotechnol. 2019;37:852–7. 31341288 10.1038/s41587-019-0209-9 PMC7015180

[36] Callahan BJ, et al. DADA2: high-resolution sample inference from Illumina amplicon data. Nat Methods. 2016;13:581–3. 27214047 10.1038/nmeth.3869 PMC4927377

[37] Quast C, et al. The SILVA ribosomal RNA gene database project: improved data processing and web-based tools. Nucl Acids Res. 2013;41:590–6. 10.1093/nar/gks1219 PMC3531112 23193283

[38] McMurdie PJ, Holmes S. Phyloseq: an R package for reproducible interactive analysis and graphics of microbiome census data. PLoS ONE. 2013;8:e61217. 23630581 10.1371/journal.pone.0061217 PMC3632530

[39] Prjibelski A, Antipov D, Meleshko D, Lapidus A, Korobeynikov A. Using SPAdes de novo assembler. Curr Protoc Bioinform. 2020;70:1–29. 10.1002/cpbi.102 32559359

[40] Wu YW, Simmons BA, Singer SW. MaxBin 2.0: an automated binning algorithm to recover genomes from multiple metagenomic datasets. Bioinformatics. 2016;32:605–7. 26515820 10.1093/bioinformatics/btv638

[41] Sieber CMK, et al. Recovery of genomes from metagenomes via a dereplication, aggregation and scoring strategy. Nat Microbiol. 2018;3:836–43. 29807988 10.1038/s41564-018-0171-1 PMC6786971

[42] Kang DD, et al. MetaBAT 2: an adaptive binning algorithm for robust and efficient genome reconstruction from metagenome assemblies. PeerJ. 2019;2019:1–13. 10.7717/peerj.7359 PMC6662567 31388474

[43] Nissen JN, et al. Improved metagenome binning and assembly using deep variational autoencoders. Nat Biotechnol. 2021;39:555–60. 33398153 10.1038/s41587-020-00777-4

[44] Kieser S, Brown J, Zdobnov EM, Trajkovski M, McCue LA. ATLAS: a snakemake workflow for assembly, annotation, and genomic binning of metagenome sequence data. BMC Bioinf. 2020;21:1–8. 10.1186/s12859-020-03585-4 PMC7310028 32571209

[45] Chklovski A, Parks DH, Woodcroft BJ, Tyson GW. CheckM2: a rapid, scalable and accurate tool for assessing microbial genome quality using machine learning. *bioRxiv* (2022). 10.1101/2022.07.11.499243 10.1038/s41592-023-01940-w 37500759

[46] Gurevich A, Saveliev V, Vyahhi N, Tesler G. QUAST: quality assessment tool for genome assemblies. Bioinformatics. 2013;29:1072–5. 23422339 10.1093/bioinformatics/btt086 PMC3624806

[47] Olson RD, et al. Introducing the bacterial and viral bioinformatics resource center (BV-BRC): a resource combining PATRIC IRD and ViPR. Nucl Acids Res. 2023;51:D678–89. 36350631 10.1093/nar/gkac1003 PMC9825582

[48] Altschul SF. BLAST algorithm. In: Encyclopedia of life sciences. New York: Willey; 2005. p. 1–4.

[49] Boratyn GM, et al. BLAST: a more efficient report with usability improvements. Nucl Acids Res. 2013;41:29–33. 10.1093/nar/gkt282 PMC3692093 23609542

[50] O’Leary NA, et al. Reference sequence (RefSeq) database at NCBI: current status, taxonomic expansion, and functional annotation. Nucl Acids Res. 2016;44:D733–45. 26553804 10.1093/nar/gkv1189 PMC4702849

[51] Pritchard L, Glover RH, Humphris S, Elphinstone JG, Toth IK. Genomics and taxonomy in diagnostics for food security: soft-rotting enterobacterial plant pathogens. Anal Methods. 2016;8:12–24. 10.1039/C5AY02550H

[52] Olmsted CN, et al. Environmental predictors of electroactive bacterioplankton in small boreal lakes. Environ Microbiol. 2023;25:705–20. 36529539 10.1111/1462-2920.16314

[53] Paysan-Lafosse T, et al. InterPro in 2022. Nucl Acids Res. 2023;51:D418–27. 36350672 10.1093/nar/gkac993 PMC9825450

[54] Davis JJ, et al. PATtyFams: Protein families for the microbial genomes in the PATRIC database. Front Microbiol. 2016;7:1–12. 26903996 10.3389/fmicb.2016.00118 PMC4744870

[55] Edgar RC. MUSCLE: Multiple sequence alignment with high accuracy and high throughput. Nucl Acids Res. 2004;32:1792–7. 15034147 10.1093/nar/gkh340 PMC390337

[56] Stamatakis A, Hoover P, Rougemont J. A rapid bootstrap algorithm for the RAxML web servers. Syst Biol. 2008;57:758–71. 18853362 10.1080/10635150802429642

[57] Chistoserdova L. Methylotrophs in natural habitats: current insights through metagenomics. Appl Microbiol Biotechnol. 2015;99:5763–79. 26051673 10.1007/s00253-015-6713-z

[58] Dumont MG, Pommerenke B, Casper P. Using stable isotope probing to obtain a targeted metatranscriptome of aerobic methanotrophs in lake sediment. Environ Microbiol Rep. 2013;5:757–64. 24115627 10.1111/1758-2229.12078

[59] He R, et al. Metabolic flexibility of aerobic methanotrophs under anoxic conditions in Arctic lake sediments. ISME J. 2021;16:1–13. 10.1038/s41396-021-01049-y. 34244610 10.1038/s41396-021-01049-y PMC8692461

[60] Rissanen AJ, et al. Vertical stratification patterns of methanotrophs and their genetic controllers in water columns of oxygen-stratified boreal lakes. FEMS Microbiol Ecol. 2021;97:1–16. 10.1093/femsec/fiaa252 PMC7840105 33316049

[61] Smith GJ, et al. Members of the genus methylobacter are inferred to account for the majority of aerobic methane oxidation in oxic soils from a freshwater wetland. MBio. 2018;9:1–17. 10.1128/mBio.00815-18 PMC6222125 30401770

[62] Islam MM, Le T, Daggumati SR, Saha R. Investigation of microbial community interactions between Lake Washington methanotrophs using genome-scale metabolic modeling. PeerJ. 2020;2020:1–28. 10.7717/peerj.9464 PMC7333651 32655999

[63] Guo W, et al. Genome-scale revealing the central metabolic network of the fast growing methanotroph *Methylomonas* sp. ZR1. World J Microbiol Biotechnol. 2021;37:1–17. 10.1007/s11274-021-02995-7 33452942

[64] Geymonat E, Ferrando L, Tarlera SE. *Methylogaea oryzae* gen., nov. sp. nov., a mesophilic methanotroph isolated from a rice paddy field. Int J Syst Evol Microbiol. 2011;61:2568–72. 21131502 10.1099/ijs.0.028274-0

[65] Zhang Y, Kitajima M, Whittle AJ, Liu WT. Benefits of genomic insights and CRISPR-Cas signatures to monitor potential pathogens across drinking water production and distribution systems. Front Microbiol. 2017;8:1–15. 29097994 10.3389/fmicb.2017.02036 PMC5654357

[66] Tavormina PL, et al. Abundance and distribution of diverse membrane-bound monooxygenase (Cu-MMO) genes within the Costa Rica oxygen minimum zone. Environ Microbiol Rep. 2013;5:414–23. 23754722 10.1111/1758-2229.12025

[67] Vekeman B, et al. Genome characteristics of two novel type I methanotrophs enriched from north sea sediments containing exclusively a lanthanide-dependent XoxF5-type methanol dehydrogenase. Microb Ecol. 2016;72:503–9. 27457652 10.1007/s00248-016-0808-7

[68] Keltjens JT, Pol A, Reimann J, Op Den Camp HJM. PQQ-dependent methanol dehydrogenases: rare-earth elements make a difference. Appl Microbiol Biotechnol. 2014;98:6163–83. 24816778 10.1007/s00253-014-5766-8

[69] Krause SMB, et al. Lanthanide-dependent cross-feeding of methane-derived carbon is linked by microbial community interactions. Proc Natl Acad Sci USA. 2017;114:358–63. 28028242 10.1073/pnas.1619871114 PMC5240692

[70] Kalyuzhnaya MG, Gomez OA, Murrell JC. The Methane-Oxidizing Bacteria (Methanotrophs). Taxonomy. Genomics Ecophysiol Hydrocarb Degrad Microbes. 2019. 10.1007/978-3-030-14796-9_10. 10.1007/978-3-030-14796-9_10

[71] Kalyuzhnaya MG, et al. Highly efficient methane biocatalysis revealed in a methanotrophic bacterium. Nat Commun. 2013;4:1–7. 10.1038/ncomms3785 24302011

[72] Stolpera DA, Revsbech NP, Canfield DE. Aerobic growth at nanomolar oxygen concentrations. Proc Natl Acad Sci USA. 2010;107:18755–60. 20974919 10.1073/pnas.1013435107 PMC2973883

[73] Chen KHC, et al. Bacteriohemerythrin bolsters the activity of the particulate methane monooxygenase (pMMO) in *Methylococcus capsulatus* (Bath). J Inorg Biochem. 2012;111:10–7. 22484247 10.1016/j.jinorgbio.2012.02.019

[74] Oshkin IY, et al. Thriving in wetlands : ecophysiology of the spiral-shaped Methanotroph *Methylospira mobilis* as revealed by the complete genome sequence. Microorganisms. 2019;7:683. 31835835 10.3390/microorganisms7120683 PMC6956133

[75] Von Canstein H, Ogawa J, Shimizu S, Lloyd JR. Secretion of flavins by Shewanella species and their role in extracellular electron transfer. Appl Environ Microbiol. 2008;74:615–23. 18065612 10.1128/AEM.01387-07 PMC2227709

[76] Coursolle D, Baron DB, Bond DR, Gralnick JA. The Mtr respiratory pathway is essential for reducing flavins and electrodes in *Shewanella oneidensis*. J Bacteriol. 2010;192:467–74. 19897659 10.1128/JB.00925-09 PMC2805334

[77] Fuller SJ, et al. Extracellular electron transport-mediated Fe(iii) reduction by a community of alkaliphilic bacteria that use flavins as electron shuttles. Appl Environ Microbiol. 2014;80:128–37. 24141133 10.1128/AEM.02282-13 PMC3910996

[78] Watanabe K, Manefield M, Lee M, Kouzuma A. Electron shuttles in biotechnology. Curr Opin Biotechnol. 2009;20:633–41. 19833503 10.1016/j.copbio.2009.09.006

[79] Scholtysik G, et al. Geochemical focusing and burial of sedimentary iron, manganese, and phosphorus during lake eutrophication. Limnol Oceanogr. 2022;67:768–83. 10.1002/lno.12019

[80] Strong PJ, Kalyuzhnaya M, Silverman J, Clarke WP. A methanotroph-based biorefinery: potential scenarios for generating multiple products from a single fermentation. Bioresour Technol. 2016;215:314–23. 27146469 10.1016/j.biortech.2016.04.099

[81] Liu LY, et al. Biological conversion of methane to polyhydroxyalkanoates: current advances, challenges, and perspectives. Environ Sci Ecotechnology. 2020;2:100029. 10.1016/j.ese.2020.100029 PMC9487992 36160923

[82] Gilman A, et al. Oxygen-limited metabolism in the methanotroph *Methylomicrobium buryatense* 5GB1C. PeerJ. 2017;5:e3945. 29062611 10.7717/peerj.3945 PMC5652258

[83] Dimitri-Kits K, Campbell DJ, Rosana AR, Stein LY. Diverse electron sources support denitrification under hypoxia in the obligate methanotroph *Methylomicrobium album* strain BG8. Front Microbiol. 2015;6:1–11. 26500622 10.3389/fmicb.2015.01072 PMC4594100

[84] Li B, et al. Iron oxides act as an alternative electron acceptor for aerobic methanotrophs in anoxic lake sediments. Water Res. 2023;234:119833. 36889095 10.1016/j.watres.2023.119833

[85] Holmes DE, Dang Y, Walker DJF, Lovley DR. The electrically conductive pili of Geobacter species are a recently evolved feature for extracellular electron transfer. Microb Genome. 2016. 10.1099/mgen.0.000072. 10.1099/mgen.0.000072 PMC5320591 28348867

