# Correction: Survival strategies of aerobic methanotrophs under hypoxia in methanogenic lake sediments

**DOI:** 10.1186/s40793-024-00609-x

**Published:** 2024-09-12

**Authors:** Almog Gafni, Maxim Rubin-Blum, Colin Murrell, Hanni Vigderovich, Werner Eckert, Nasmille Larke-Mejía, Orit Sivan

**Affiliations:** 1 Department of Earth and Environmental Sciences, Ben-Gurion University of the Negev, Beer Sheva, Israel; 2 Biology Department, National Institute of Oceanography, Israel Oceanographic and Limnological Research, Haifa, Israel; 3 School of Environmental Sciences, University of East Anglia, Norwich, UK; 4 The Yigal Allon Kinneret Limnological Laboratory, Israel Oceanographic and Limnological Research, Migdal, Israel; 5 Quadram Institute Bioscience, Norwich Research Park, Norwich, UK; 6 Department of Marine Biology, Leon H. Charney School of Marine Sciences, University of Haifa, Haifa, Israel

**Correction: Environmental Microbiome (2024) 19:44** 10.1186/s40793-024-00586-1

Following publication of the original article, the following three concerns were brought to the attention of the authors: Figure 4 was low resolution and, as a result, difficult to read; affiliation ‘6’ (see the original article) was missing from the affiliations of the second author, Maxim Rubin-Blum; the given and family names of the first author, Almog Gafni, were the wrong way around. These errors have since been corrected in the published article. The authors thank you for reading this erratum and apologize for any inconvenience caused.

