# Supplementary material for: Survival strategies of aerobic methanotrophs under hypoxia in methanogenic lake sediments
Source: Environ Microbiome. 2024 Jul 2;19:44. doi: 10.1186/s40793-024-00586-1 (PMC11218250; doi:10.1186/s40793-024-00586-1)
Supplement: Supplementary file 1 — Additional file 1. [file 40793_2024_586_MOESM1_ESM.docx]

Supplementary information:

Survival strategies of aerobic methanotrophs under hypoxia in methanogenic lake sediments

Gafni Almog^1^, Maxim Rubin-Blum^2^, J. Colin Murrell^3^, Hanni Vigderovich^1^, Werner Eckert^4^, Nasmille Larke-Mejía^5^, and Orit Sivan^1^

^1^ Department of Earth and Environmental Sciences, Ben-Gurion University of the Negev, Beer Sheva, Israel

^2^ Israel Oceanographic and Limnological Research, National Institute of Oceanography, Haifa, Israel

^3^ School of Environmental Sciences, University of East Anglia, Norwich, NR4 7TJ, UK

^4^ The Yigal Allon Kinneret Limnological Laboratory, Israel Oceanographic and Limnological Research, Migdal, Israel

^5^ Quadram Institute Bioscience, Norwich Research Park, Norwich, NR4 7UQ, UK

1. **Methods**
   1. *DNA-SIP experiment*

Supplementary Table S1 (Excel): DNA-SIP sample list, sampling time, samples used for 16S rRNA gene sequencing and metagenome analysis.

- 1. *Anoxic experiment*

Supplementary Table S2 (Excel): Anoxic experiment sample list, sampling time, samples used for 16S rRNA gene sequencing and metagenome analysis.

Gases spiking events:

| Treatment | Spiking (days) |
| --- | --- |
| 1% O_2_ + CH_4_ | 0,3,9,11,14,17,19,21,25,37 |
| 1% O_2_ + N_2_ | 0,3,9,11,14,37 |

- 1. *Bioinformatics*

Supplementary Table S3 (Excel): list of *Methylococcales* genomes used, including detailed information.

Supplementary Table S4 (Excel): list of average nucleotide identity (ANI) values between *Methylococcales* genomes.

Supplementary Table S5 (Excel): list of proteins used for phylogenetic tree construction.

1. **Results and Discussion**
   1. *Methylobacter and Methylotenera dominate the DNA-SIP experiment*

The relative abundance of the microbial community remained the same regardless of the sampling period (Figure S1a). The microbial community similarity relationship analysis, using PCoA based on Bray–Curtis dissimilarity distances, further demonstrated no difference among the ^13^C-methane labeled fractions regardless of the sampling time (Figure S2b).

| 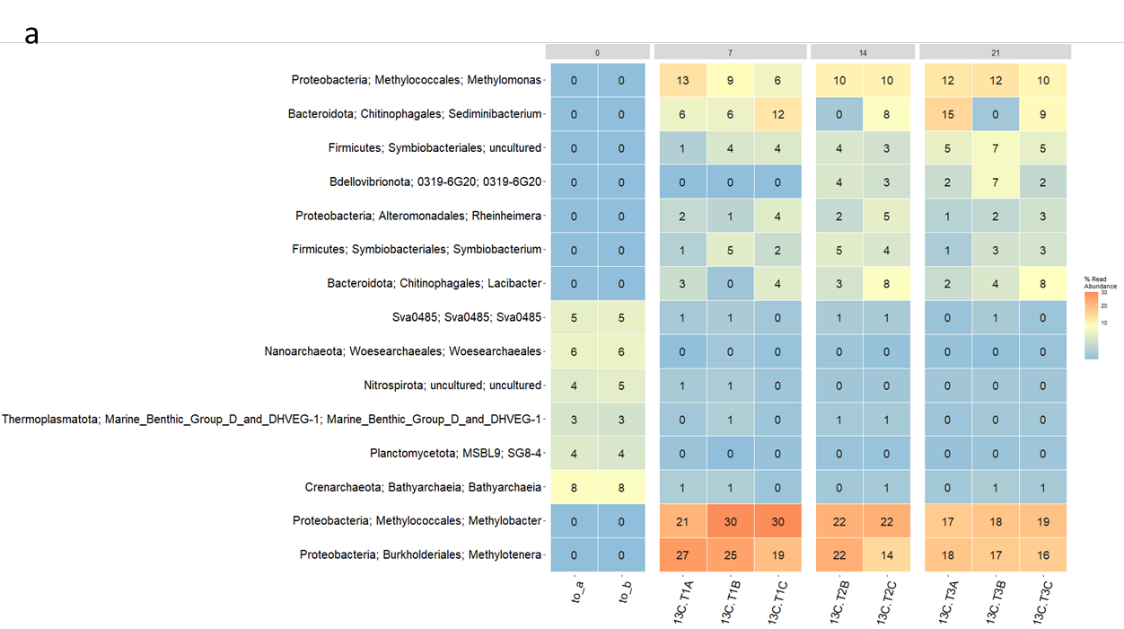 |
| --- |
| 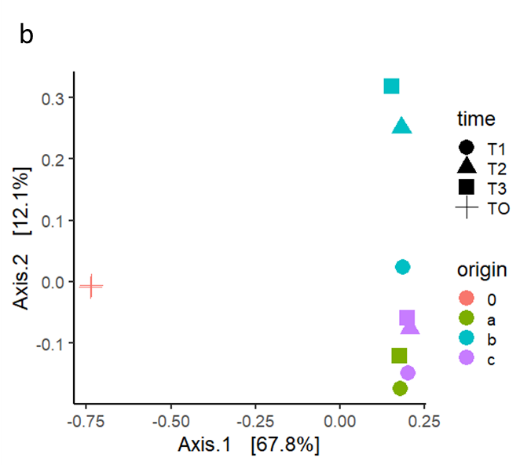 |
| Figure S1: (a) Relative abundance (%) of the top 15 genera in the labeled ^13^C-methane-fed cultures. (b) Principal component analysis of the same samples, T1, T2 and T3 represent 7, 14 and 21-day incubation (circles, triangle, square, respectively), time zero (cross). The color coded indicates the vial origin of the sample a, b or c. |

- 1. *Methylomonas and Methylotenera dominate the hypoxic experiment*

The community in T_o_ and the N_2_+O_2_ treatment differ from the CH_4_+O_2_ treatment community.

| 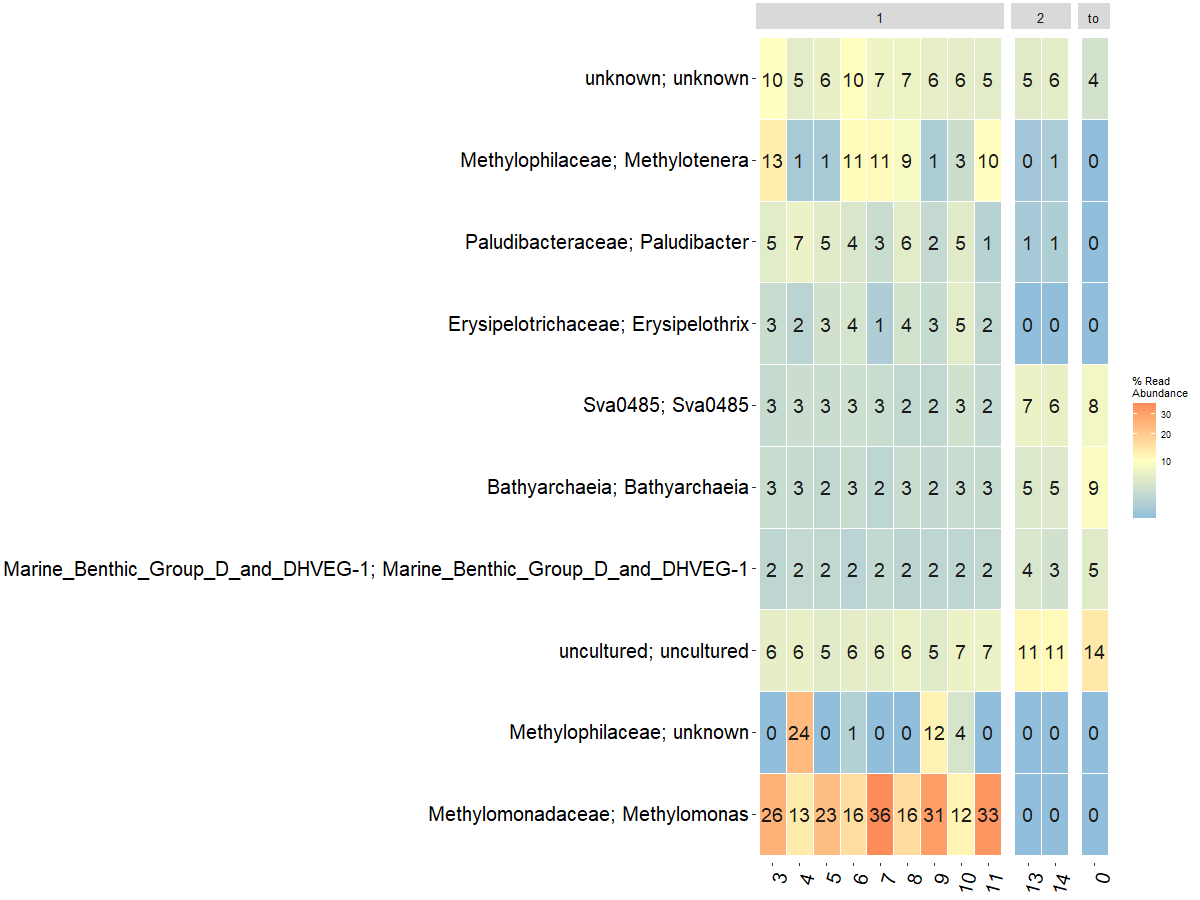 |
| --- |
| Figure S2: Relative abundance (%) of the top 10 genera in the hypoxic experiment treatment CH_4_+O_2,_ N_2_+O_2,_ and time zero sampling are marked as 1,2, and to respectively. |

- 1. *Metabolic reconstruction of the novel Lake Kinneret methanotrophs*

Supplementary Table S6 (Excel): list of predicted proteins and their copy number as predicted in LK *Methylococcales*.

- 1. *Adaptation of Methylococcales to the hypoxic environment is not unique to Lake Kinneret*

Supplementary Table S7 (Excel): list of predicted proteins used for the presence-absence list in Figure 4. This includes protein type, location, and the name for each *Methylococcales* genomes.

Supplementary Table S8 (Excel): A list of predicted OMC proteins assigned using the FEET pipeline, manually verified using the InterPro database, and used for the presence-absence list in Figure 4. This includes protein type, location, protein sequence, and the name of each *Methylococcales* genomes.

Supplementary Table S9 (Excel): Additional information of the *Methylococcales* genomes presenting a presence-absence list for the following predicted proteins: particulate methane monooxygenase *pmoCAB* operon and *pxmABC* operon, soluble methane monooxygenase (mmoXYBZDC), lanthanide-dependent methanol dehydrogenases (xoxF), and methanol dehydrogenase (mxaF).
